# Are Loneliness and Social Isolation Equal Threats to Health and Well-being? An Outcome Wide Longitudinal Approach

**DOI:** 10.1093/geroni/igab046.3359

**Published:** 2021-12-17

**Authors:** Tatiana Henriksson, Julia Nakamura, Eric Kim

**Affiliations:** 1 University of British Columbia, Vancouver, British Columbia, Canada; 2 University of British Columbia, University of British Columbia/Vancouver, British Columbia, Canada

## Abstract

The detrimental effects of loneliness and social isolation on health and well-being outcomes are well documented. In response, governments, corporations, and community-based organizations have begun leveraging emerging tools to create interventions and policies aimed at reducing loneliness and social isolation at-scale. However, these efforts are frequently hampered by a key knowledge gap: when attempting to alleviate specific health and well-being outcomes, decision-makers are unsure whether to target loneliness, social isolation, or both. Participants (N=13,752) were from the Health and Retirement Study- a diverse nationally representative, and longitudinal sample of U.S. adults aged > 50 years. We examined how changes in loneliness and social isolation over a 4-year follow-up period (from t0:2008/2010 to t1:2012/2014) were associated with 32 indicators of physical-, behavioral-, and psychosocial-health outcomes 4-years later (t2:2016/2018). We used, multiple logistic-, linear-, and generalized-linear regression models, and adjusted for sociodemographics, personality traits, pre-baseline levels of both exposures (loneliness and social isolation), and all outcomes (t0:2008/2010). After adjusting for a wide range of covariates, we observed that both loneliness and social isolation have similar effects on physical health outcomes and health behaviors, whereas loneliness is a stronger predictor of psychological outcomes. In particular, behavioral dimensions of the social isolation measure (i.e., participation in social/religious activities, social interaction frequency) were most strongly associated with the largest number of health and well-being outcomes, including all-cause mortality. Loneliness and social isolation have independent effects on various health and well-being outcomes, thus, should be distinct targets for interventions aimed at improving the health and well-being.

